# Multiplexed Nanopore Sequencing of HLA-B Locus in Māori and Pacific Island Samples

**DOI:** 10.3389/fgene.2018.00152

**Published:** 2018-04-30

**Authors:** Kim N. T. Ton, Simone L. Cree, Sabine J. Gronert-Sum, Tony R. Merriman, Lisa K. Stamp, Martin A. Kennedy

**Affiliations:** ^1^Department of Pathology and Biomedical Science, University of Otago, Christchurch, New Zealand; ^2^JSI Medical Systems GmbH, Ettenheim, Germany; ^3^Biochemistry Department, University of Otago, Dunedin, New Zealand; ^4^Department of Medicine, University of Otago, Christchurch, New Zealand

**Keywords:** HLA-B, nanopore sequencing, Māori, Pacific Island, pharmacogenetics, Polynesian

## Abstract

The human leukocyte antigen (HLA) system encodes the human major histocompatibility complex (MHC). HLA-B is the most polymorphic gene in the MHC class I region and many HLA-B alleles have been associated with adverse drug reactions (ADRs) and disease susceptibility. The frequency of such HLA-B alleles varies by ethnicity, and therefore it is important to understand the prevalence of such alleles in different population groups. Research into HLA involvement in ADRs would be facilitated by improved methods for genotyping key HLA-B alleles. Here, we describe an approach to HLA-B typing using next generation sequencing (NGS) on the MinION™ nanopore sequencer, combined with data analysis with the SeqNext-HLA software package. The nanopore sequencer offers the advantages of long-read capability and single molecule reads, which can facilitate effective haplotyping. We developed this method using reference samples as well as individuals of New Zealand Māori or Pacific Island descent, because HLA-B diversity in these populations is not well understood. We demonstrate here that nanopore sequencing of barcoded, pooled, 943 bp polymerase chain reaction (PCR) amplicons of 49 DNA samples generated ample read depth for all samples. HLA-B alleles were assigned to all samples at high-resolution with very little ambiguity. Our method is a scaleable and efficient approach for genotyping HLA-B and potentially any other HLA locus. Finally, we report our findings on HLA-B genotypes of this cohort, which adds to our understanding of HLA-B allele frequencies among Māori and Pacific Island people.

## Introduction

The human leukocyte antigen (HLA) locus contains a large family of genes encoding the human major histocompatibility complex (MHC) proteins. It is located on chromosome 6p21 and divided into three classes: class I, class II, and class III. HLA molecules are extremely variable due to their peptide-binding function and are associated with autoimmune diseases and adverse drug reactions (ADRs) (Tiwari and Terasaki, 1985; Bharadwaj et al., 2010). HLA-B is the most polymorphic gene, with over 4,600 known alleles encoding 3,408 unique proteins (IMGT/HLA Database release 3.27 in January 2017) (Robinson et al., 2014). Previous studies have identified particular HLA-B alleles as risk factors for drug-induced hypersensitivity reactions (Alfirevic and Pirmohamed, 2010; Sukasem et al., 2014). For example, screening for the HLA-B^*^57:01 allele is recommended prior to abacavir treatment to decrease risk of a hypersensitivity reaction (Martin et al., 2012). Strong association between HLA-B^*^58:01 and allopurinol-induced severe cutaneous adverse reactions such as Stevens–Johnson syndrome (SJS) or toxic epidermal necrolysis (TEN) have been reported across different populations (Somkrua et al., 2011). However, some HLA alleles associated with drug-induced hypersensitivity can be ethnic group-specific. For example, HLA-B^*^15:02 allele is a risk factor for carbamazepine-induced SJS/TEN found in several Asian populations but not in Caucasian and Japanese populations (Tassaneeyakul et al., 2010; Phillips et al., 2018). Given the correlation of ethnic-specific risk alleles with ADRs, a good knowledge of HLA allele frequencies, and the prevalence of susceptibility alleles in particular, is important for the study of pharmacogenetics.

The high level of polymorphism in the MHC family means HLA genotyping is complex. HLA alleles are mostly determined by the sequences of exons 2 and 3 in HLA class I genes and exon 2 in HLA class II genes (Shiina et al., 2012). Present DNA-based methods for HLA typing are polymerase chain reaction (PCR) -sequence-specific priming (PCR-SSP), PCR-sequence-specific oligo hybridization (PCR-SSO), PCR-restriction fragment length polymorphism (PCR-RFLP), and sequence-based typing (SBT) (Tait et al., 2009; Bontadini, 2012; Erlich, 2012). SBT is currently considered the gold standard method applied in high-resolution HLA typing (Erlich, 2012). However, this approach may generate ambiguous HLA typing due to haplotype phase issues and incomplete sequencing. Other approaches have various limitations in resolution, workflow complexity, probe design and testing requirements, as new HLA alleles are submitted to the IMGT/HLA sequence database (Erlich, 2012; Shiina et al., 2012).

Recently, next-generation sequencing (NGS) methods have become widely established for HLA typing (Abbott et al., 2006; Bentley et al., 2009; Erlich et al., 2011; Erlich, 2012; Shiina et al., 2012; Hosomichi et al., 2013, 2015; Schöfl et al., 2017). Such approaches reduce the risk of phase ambiguity and allow high-throughput, high-resolution HLA typing. Various approaches to HLA typing using NGS have been developed, including PCR-based HLA sequencing (Erlich et al., 2011; Boegel et al., 2012; Liu et al., 2012; Shiina et al., 2012; Hosomichi et al., 2013; Schöfl et al., 2017), whole exome sequencing (WES) or whole genome sequencing (WGS) data-derived typing (Liu et al., 2012; Major et al., 2013). However, these methods are of limited value for research studies of ADRs, where a more targeted screening for specific HLA alleles may be all that is required.

Here we describe the development of high-throughput HLA typing from next-generation DNA sequencing data, focusing on the HLA-B locus, and its application to identifying HLA-B alleles within the Māori and Pacific Island population of New Zealand. Our strategy took advantage of a recent iteration of the novel NGS platform, the MinION™ nanopore sequencer (Oxford Nanopore Technologies), and barcode sequences for labeling and simultaneously analyzing HLA-B amplicons from multiple samples. The MinION is a tiny, portable nanopore sequencer powered by a USB 3.0 port (Quick et al., 2014). It allows analysis of sequencing data in real time and generation of very long reads (Urban et al., 2015). A small pilot study used the device to examine HLA-A and HLA-B alleles from a single sample, using the earlier, quite error-prone R7.0 flow-cell chemistry (Ammar et al., 2015). Oxford Technologies released a new chemistry (R9.4) in May 2016, which has proven to be more accurate and with higher throughput. This major update motivated us to examine the performance of this pocket-sized device on one of the most polymorphic genes in the human body, HLA-B.

New Zealand is a multi-ethnic country with people from many different nations. The Māori are the indigenous Polynesian people, who first settled in New Zealand. New Zealand is also home to many people from the Pacific Islands, with its main city of Auckland referred to as the “Polynesian capital of the world” (Anae, 2005). Polynesian people in New Zealand include people from Samoa, Cook Islands, Tongan, Fiji, Niue and Tokelau, which together account for 7.6% of New Zealand population (Geck, 2017). To date, there is a paucity of studies providing prevalence data of HLA-B alleles in Māori and Pacific Island population in New Zealand (Abbott et al., 2006; Edinur et al., 2013). Given that HLA-B alleles are so relevant to disease predisposition and ADRs, it is important to establish a prevalence dataset for HLA-B for these population groups. Therefore, we sought to develop an assay for HLA-B screening in this population, using the MinION.

## Materials and methods

### Participants

Forty unrelated Māori and Pacific Island individuals with no history of inflammatory disorders were recruited from the Otago and Auckland regions of New Zealand. The proportion of ancestry for each participant was estimated by recording the ethnicity of all four grandparents. This study was carried out in accordance with the recommendations of the Standard Operating Procedures for Health and Disability Ethics Committees (New Zealand), as reviewed by the Lower South Ethics Committee (New Zealand), with written informed consent from all subjects. Additionally, five individuals were included from a local study on ADRs called Understanding ADRs or responses Using Genome Sequencing (UDRUGS) (Maggo et al., 2017), which was approved by the Southern Health and Disability Ethics Committee (New Zealand), with written informed consent from all subjects. A further four samples of known HLA-B genotype were obtained from the Coriell Institute for Medical Research (Camden, NJ, USA). These nine individuals were used as a reference set for the MinION analysis, after confirmation by either Sanger sequence based typing (SBT) or data retrieved from the 1000 Genomes project, or both.

### HLA-B genotyping by Sanger sequencing

We selected a subset of our participants (four Polynesian, five UDRUGS and two Coriell samples) to analyze by Sanger sequencing, as additional references. Nested PCR was used to amplify a 1,710 bp region spanning exon 2 and exon 3 of HLA-B. PCR products were diluted and used as templates in second round PCR to amplify a 943 bp amplicon. These amplicons were then directly sequenced in both forward and reverse directions using a set of six sequencing primers (Table 1). The primers used for amplification included some nucleotide redundancies at sites of HLA-B variation, to prevent allelic drop-out during the PCR step. All PCR primers and sequencing primers were derived from published work (Abbott et al., 2006; Cotton et al., 2012). The HLA-B genotypes of these 11 samples were generated from the Sanger sequence data using SBTengine v.3.10.0.2610 (GenDX, Utrecht, Holland).

**Table 1:** Primers used for amplifying and sequencing.

**Assay**	**Primer**	**Sequence**	**Source**
SBT	HLA-B_1710_F	TGTCGGGTCCTTCTTCCAGG	Abbott et al., 2006
	HLA-B_1710_R	GAAAATTCAGGCGCTTT	
	HLA-B_943_F	GCAGGCGGGGGCGCAGGACC	Cotton et al., 2012
	HLA-B_943_R	GGAGATGGGGAAGGCTCCCCACT	
	HLA-B_Seq1	GGAGCCGCGCCGGGAGGAGGGTC	
	HLA-B_Seq2	GGATGGGGAGTCGTGACCT	
	HLA-B_Seq3	ACKGKGCTGACCGCGGGG	
	HLA-B_Seq4	CGGGGTCACTCACCGKCCTC	
	HLA-B_Seq5	GGSCKGGGCCAGGGTCTCAC	
	HLA-B_Seq6	ACTGCCCCTGGTACCMGCGC	
MinION	HLA-B_MinION_F	TTTCTGTTGGTGCTGATATTGCGGGAGGAGMRAGGGGACCSCAG	
	HLA-B_MinION_R	ACTTGCCTGTCGCTCTATCTTCGGAGGCCATCCCCGGCGACCTAT	

*Underlined letters are IUPAC codes indicating base redundancies at positions corresponding to known HLA-B variants*.

### Minion library construction

The primer used to amplify a fragment of 943 bp HLA-B exon 2 and 3 included a specific sequence (Table 1) at the 5′ end, which is compatible with barcode sequences (Oxford Nanopore Technologies). A standard protocol of the Kapa LongRange Hotstart DNA PCR (Kapa Biosystems) was applied, consisting of 1X Kapa LongRange Buffer (without Mg^2+^), 2.0 mM MgCl_2_, 0.3 mM dNTPs (2.5 mM each dNTP), 0.5 μM of each primer, 1.25 U/50 μl Kapa LongRange HotStart DNA Polymerase, 50 ng genomic DNA, and water up to 50 μl. Thermal cycling conditions were 94°C for 3 min, 25 cycles at 94°C for 15 s, 68°C for 15 s, and 72°C for 1 min, with a final extension at 72°C for 1 min. The PCR products were visualized by electrophoresis on 2% agarose gel stained with SYBR™ Safe DNA Gel Stain (Invitrogen), and then purified using 1x Agencourt AMPure XP beads (Beckman Coulter).

PCR products were quantified by Qubit® 2.0 Fluorometer (ThermoFisher Scientific) and were diluted to 2 nM in water. A second PCR was performed to incorporate barcode sequences using Oxford Nanopore PCR Barcoding kit (EXP-PBC096). Each 100 μl reaction contained 1X Kapa LongRange Buffer (without Mg^2+^), 2.0 mM MgCl_2_, 0.3 mM dNTPs (10 mM each dNTP), 0.2 μM PCR Barcode primers (from BC01 to BC49), 2.0 U Kapa LongRange HotStart DNA Polymerase, and 0.5 nM of first-round PCR product. The cycling parameters were an initial denaturation 95°C for 3 min, followed by15 cycles at 95°C for 15 s, 62°C for 15 s, and 65°C for 1 min, with a final extension at 65°C for 1 min. All 49 barcoded products were cleaned up with 1x Agencourt AMPure XP beads, then quantified. Purified PCR products were normalized by concentration before being pooled for library preparation.

The pooled library was prepared using the Oxford Nanopore Sequencing protocol (SQK-NSK007). We used 5 μg of library as an input, instead of the recommended 1 μg, to improve yield for downstream steps. Briefly, 5 μg of purified amplicon library was prepared with the NEBNext end repair module (New England Biolabs), then dA-tailed using the NEBNext dA-tailing module (New England Biolabs). The end-prepared, dA-tailed library was subsequently ligated with leader and hairpin adapters, followed by purification using Dynabeads® MyOne™ Streptavidin C1 beads (Invitrogen).

The final prepared library from 49 participants was loaded into the MinION R9.4 flowcell (Oxford Nanopore Technologies). The flowcell was run for 48 h using the MinKNOW software (0.51.1.39).

### Data analysis

Raw sequence data were uploaded for base-calling using Metrichor software (2D Basecalling for SQKMAP007 - v1.107). Sequences in FASTA format were extracted from the raw FAST5 files using poretools v.0.6.0 (Loman and Quinlan, 2014). Statistical analysis of the MinION sequencing data were generated and visualized by De Coster et al. (2017). In order to determine error rates, base-calls in FASTQ format were extracted using poretools v.0.6.0 (Loman and Quinlan, 2014) and then aligned against Sanger sequenced reference using BWA-MEM (version 0.7.12-41044), parameter “-x ont2d”. Additional statistical analyses were performed with Python and R scripts available at https://github.com/camilla-ip/marcp2. We only used two-dimensional (2D) reads, which are consensus calls of the combined template and complement strands, to perform HLA-B locus high-resolution typing with SeqPilot v.4.3.1 using default settings (JSI medical systems). HLA-B ambiguities were designated as G group nomenclature (http://hla.alleles.org/alleles/g_groups.html). Samples that could not be assigned genotypes due to mismatches were re-analyzed for error correction. Nanopolish pipeline was applied on input reads of these samples to check improvement on the base-level accuracy (Loman et al., 2015). After polishing, consensus sequences were re-processed to assign HLA-B genotypes of these samples.

## Results

A total of 40 Maori and Pacific Island, four Coriell and five UDRUGS individuals were selected for library construction. We successfully amplified a region of 943 bp spanning exon 2 and 3 of HLA-B in a single PCR. After purifying, all 49 PCR products were diluted to reach the desired concentration (2.0 nM). Three gave insufficient yield, ranging from 0.19 to 1.47 nM. However, these three were still included to test whether they could be effectively amplified in the barcoding step.

**Figure 1:**
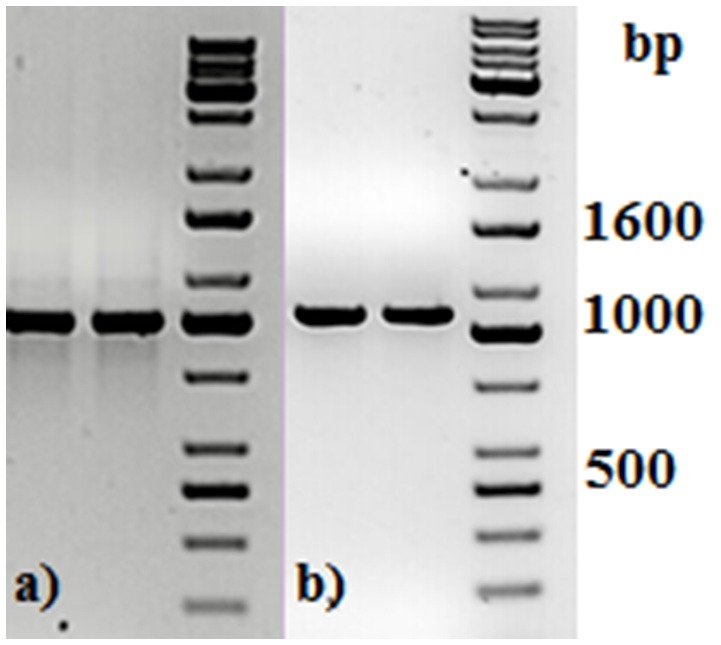
Agarose gel electrophoresis of two representative PCR amplicons **(a)** before and **(b)** after PCR barcoding step. DNA molecular size marker obtained from KAPA Universal Ladder (KAPA Biosystems, Boston, MA).

Two primers used in the first PCR were tailed with the adapter sequences, which were compatible with Oxford Nanopore barcodes. Each PCR product was then amplified with barcode primers, at which point all 49 PCR products were tagged with barcodes, increasing their length to 1,063 bp (Figure 1).

PCR products were subjected to normalization prior to pooling and sequencing on the MinION (~368 ng/each). Five samples had significantly lower concentrations than other samples (range: 20.4–66.8 ng), but these were included in the pool (Table 2). The total DNA quantity in the pool was 7.5 μg and 5 μg was used for downstream steps. After end-repair, adapter ligation and purification steps, 585 ng of prepared library remained and was loaded into the MinION flow cell.

**Table 2:** DNA quantity and number of mapped reads of poorly amplified samples.

**Individual**	**Index**	**DNA quantity (ng)**	**Mapped 2D read (number)**
PI_G2	BC15	66.84	473
PI_C3	BC19	20.4	80
PI_D3	BC20	57.96	869
PI_H3	BC22	64.44	333
PI_C6	BC42	33.36	158

**Figure 2:**
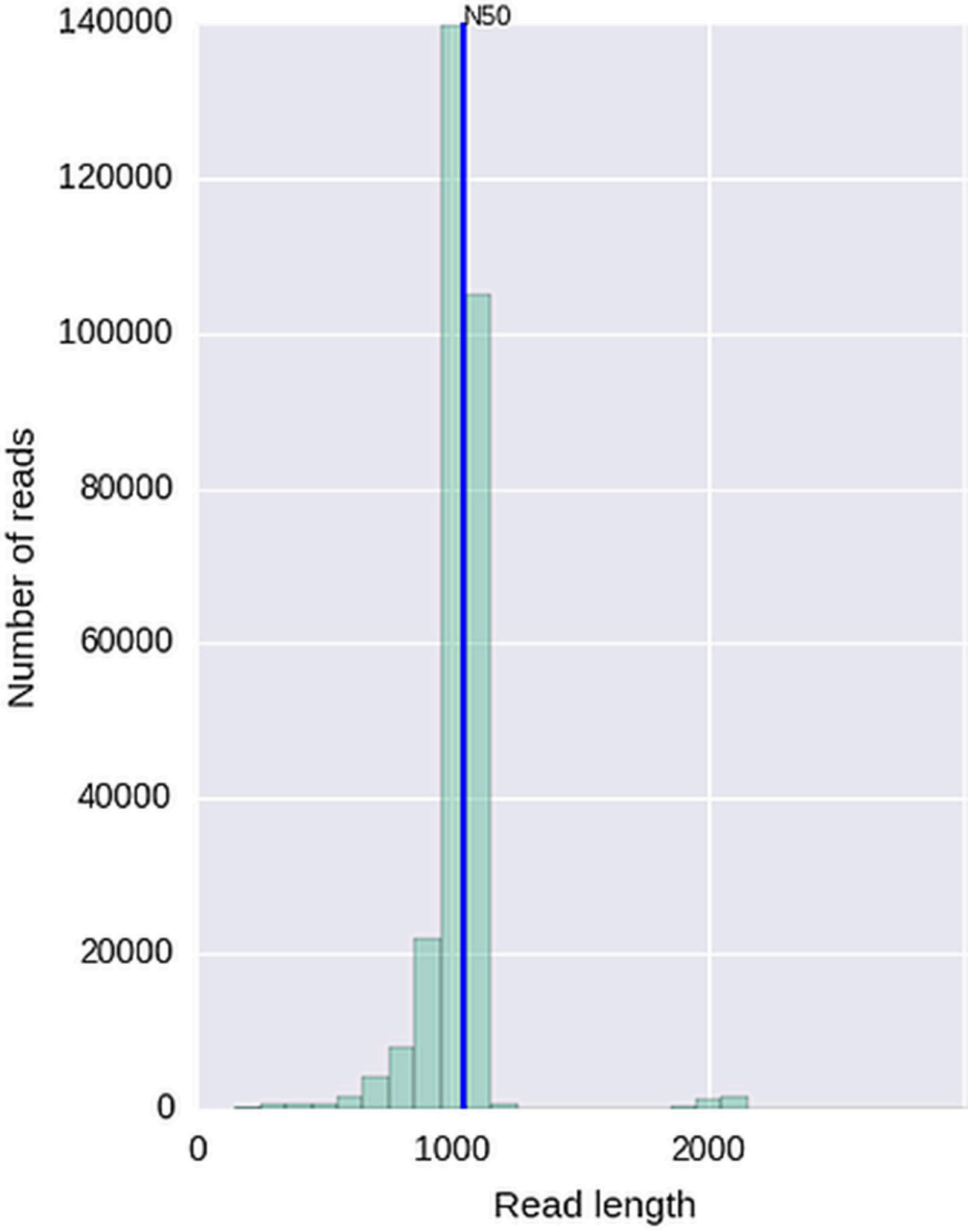
Histogram of read length with read N50 metric.

**Figure 3:**
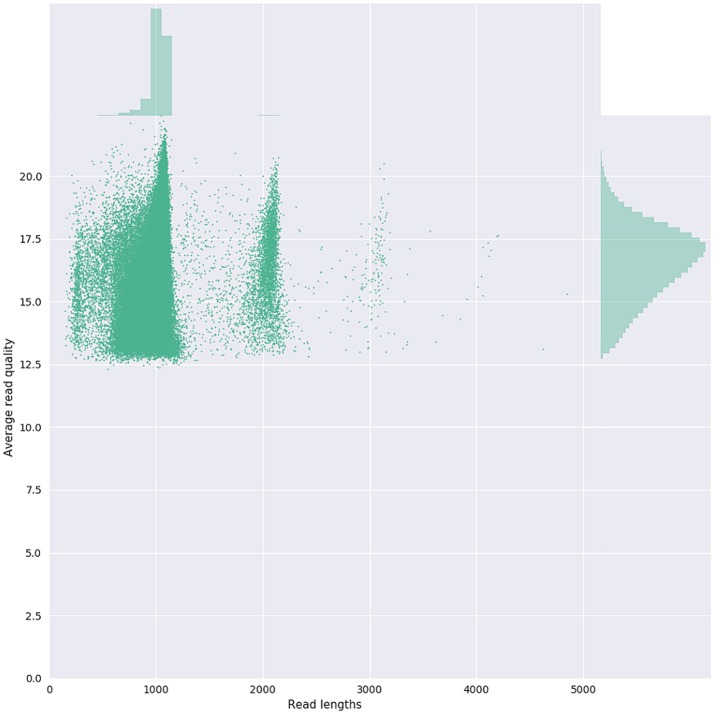
Bivariate plot shows with a kernel density estimate the read length compared to the average read basecall Phred quality.

**Figure 4:**
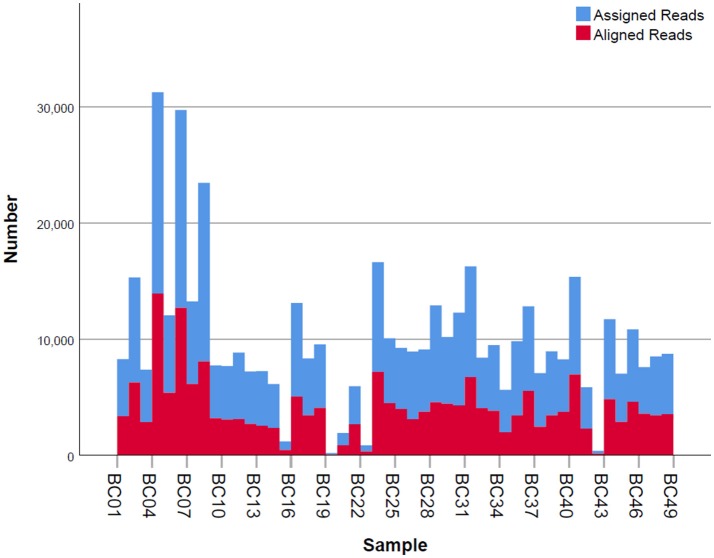
Number of mapped and unmapped reads per individual. Blue bars indicate the assigned reads that are mapped to the regions of interest. Red bars are the aligned reads that pass the filter and are used for allele assignment.

Given that for this version of the MinION chemistry, 2D reads were more accurate and had greater length than 1D reads, we extracted only the 2D reads for downstream analysis. After conversion, all 49 FASTQ files were imported into SeqPilot software for HLA-B allele assignment. The mean read depth was 5,807x and the mean read length was 1,029 bp, close to the expected size of all amplicons (Figure 2). An average of 5,854 sequence reads per barcoded sample was obtained from a total of 289,095 2D pass reads. The proportion of reads with a *Q*-value threshold of 15 was 83.3% (Figure 3). There were 286,852 reads with uniquely identified barcodes, of which 199,297 reads passed the quality filters and were aligned to the assigned allele sequences. The distribution histogram of both assigned and aligned reads for each barcoded sample is shown in Figure 4.

The five samples that amplified poorly still produced ample reads for SeqPilot to generate HLA-B typing calls (Table 2). Notably, individual PI_C3, which had the lowest number of mapped reads (80) that aligned to the reference sequence, was assigned the alleles HLA-B^*^44:04, 56:02:01. We are confident these alleles are correct as no mismatches occurred at key polymorphic sites in the reads.

**Figure 5:**
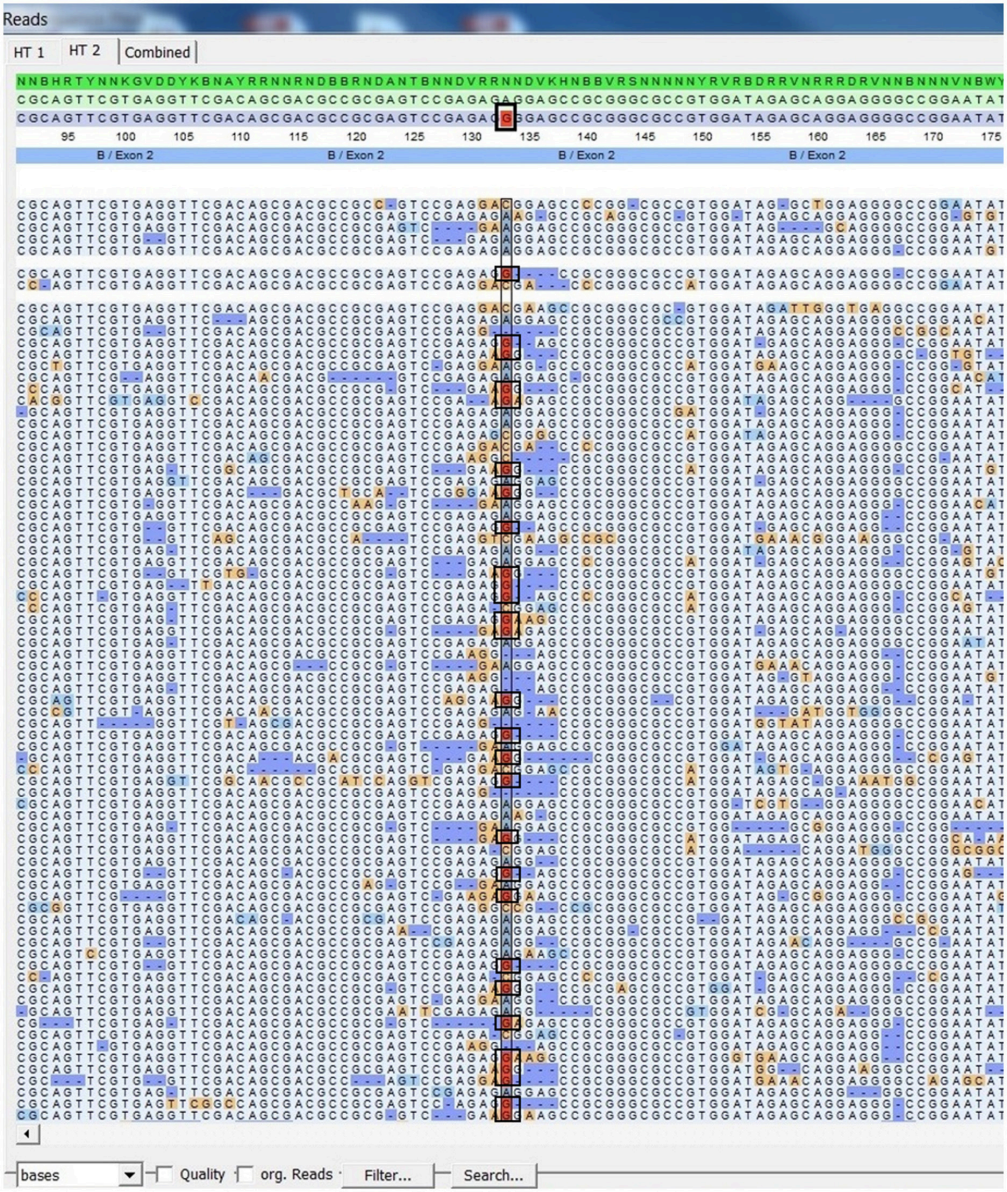
SeqPilot screenshot from mapping sequencing reads with reference sequences. Bias phasing position (black box) followed by deletion errors.

**Figure 6:**
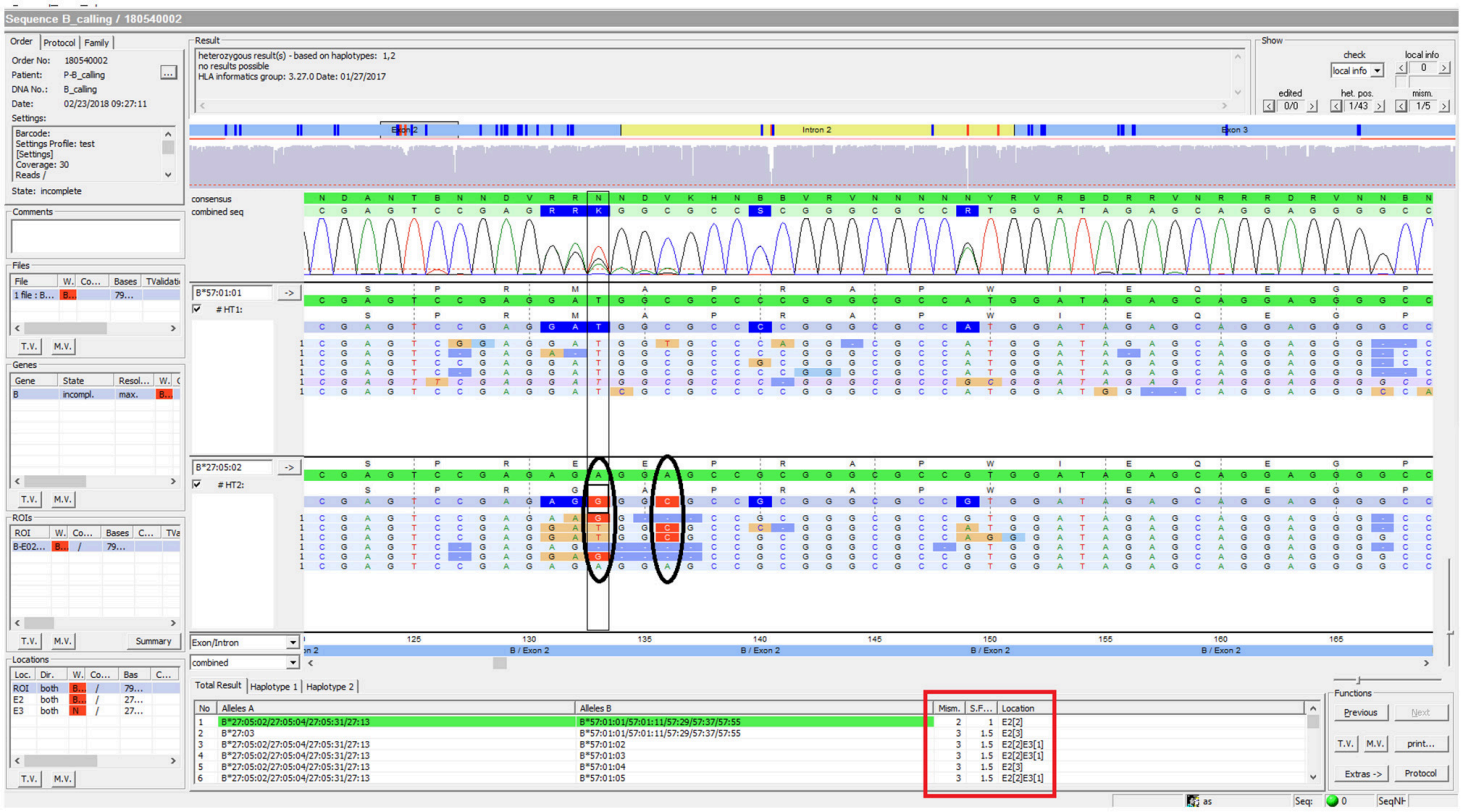
SeqPilot screenshot from analysis of sample PI_A2. This illustrates the ambiguous results of sample PI_A2 with several possible alleles and their corresponding mismatched locations. HLA-B^*^27:05:02G was not confidently called due to two mismatches at nucleotide position 133 and 136 of exon 2. The software provides possible allele pairs with number of mismatches and their corresponding positions (red box). The mismatched nucleotides from the assigned allele are shown in each sequence read (ellipses).

**Figure 7:**
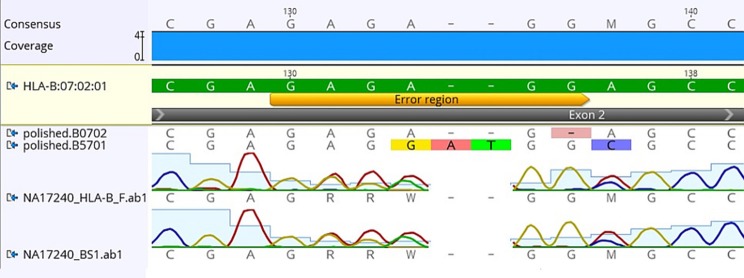
Comparison of consensus sequences of NA17240 (BC47) obtained from Sanger sequencing with those from Nanopolish. Yellow bar indicates the region where mismatched alignment happens (nt 130-136). HLA-B^*^07:02:01 is the reference sequence; polished.B0702 and polished B5701 are consensus sequences generated by Nanopolish. Data was visualized on Geneious v.9.1.5 (Biomatters Ltd., New Zealand).

Using FASTQ files as inputs, SeqPilot effectively assigned HLA-B alleles of 49 individuals. There were 4 homozygotes and 45 heterozygotes, resulting in 38 alleles called at the third field (formerly 6-digit) resolution and five alleles at second field (4-digit) resolution (Table 3). There were six that could not be automatically called by the software due to mismatches with reference sequences. Notably, the phasing bias mostly happened at nucleotide position 130–136 of exon 2. We realized that there was A/G heterozygous at nucleotide 133 and the sequence around this position was a repeat of G and A (GAGAGRGGAG). We also observed that if nucleotide 133 was G, there was usually a deletion of two to four nucleotides (Figure 5). Figure 6 illustrates the ambiguous results of sample PI_A2 with several possible alleles and their corresponding mismatched locations. HLA-B^*^27:05:02G was not able to be confidently called due to two mismatches at the area mentioned above (Figure 6). Sanger sequencing these six samples confirmed that these mismatches were not PCR artifacts, suggesting these errors might happen during the nanopore sequencing step. To explore whether such errors could be corrected, we ran the Nanopolish pipeline to compute consensus sequences with improved base quality. After comparing these polished sequences with sequences obtained from Sanger sequencing, we found that Nanopolish did not improve the accuracy for these error regions further (Figure 7). In this case, we manually assigned allele pairs from the suggested list, choosing those with the least mismatch. Although other NGS approaches have been applied to HLA analysis, Sanger sequencing is regarded as the gold standard for HLA-typing. Consequently, we selected 11 individuals for HLA-B genotyping using the Sanger SBT method for validation. These were four Polynesian, five UDRUGS and two Coriell samples. Six amplicons covering exon 2 and 3 of each individual were directly sequenced. HLA-B alleles were defined based on these polymorphic sequences. Using SBTengine (GenDX, Utrecht, Holland) for analysis, we found that there was a high consistency of variant calls with calls derived from the MinION data. Our genotyping data for all four Coriell individuals were concordant with the data from these samples generated by the 1000 Genomes project. These results indicate that this MinION sequencing method is able to generate consensus sequences for high-resolution HLA-B typing with considerable accuracy.

**Table 3:** Assignment result of HLA-B obtained from MinION sequencing and SBT.

**Sample**	**Ethnicity**^#^	**MinION**		**SBT**	
NA16688^§^	Ch	B^*^15:07:01G	^*^35:01:01G		
NA17019^¥^	Ch	B^*^15:02:01G	^*^15:11:01G	B^*^15:02:01G	^*^15:11:01G
NA17240^¥^^§^	C	B^*^07:02:01G	^*^57:01:01G	B^*^07:02:01G	^*^57:01:01G
NA19834^¥^	A	B^*^35:01:01G	^*^39:10:01		
UDRUGS01^¥^	C	B^*^40:02:01G	^*^44:02:01G	B^*^40:02:01G	^*^44:02:01G
UDRUGS02^¥^	C	B^*^15:01:01G	^*^44:02:01G	B^*^15:01:01G	^*^44:02:01G
UDRUGS29^¥^^§^	C	B^*^18:01:01G	^*^27:09	B^*^18:01:01G	^*^27:09
UDRUGS41^¥^	C	B^*^15:01:01G	^*^51:01:01G	B^*^15:01:01G	^*^51:01:01G
UDRUGS44^¥^	C	B^*^15:01:01G	^*^44:03:01G	B^*^15:01:01G	^*^44:03:01G
PI_A1^¥^	0.25 NM 0.75 C	B^*^37:01:01G	^*^39:01:01G	B^*^37:01:01G	^*^39:01:01G
PI_A2^¥^^§^	0.5 NM 0.5 C	B^*^27:05:02G	^*^57:01:01G	B^*^27:05:02G	^*^57:01:01G
PI_A3^¥^	0.5 CM 0.5 P	B^*^39:05:01	^*^52:01:01G	B^*^39:05:01	^*^52:01:01G
PI_A5	0.125 NM 0.25 P 0.625 C	B^*^08:01:01G	^*^35:01:01G		
PI_B1	1.0 S	B^*^46:01:01G	^*^55:02:01G		
PI_B2	0.5 NM 0.5 C	B^*^07:02:01G	^*^57:01:01G		
PI_B3	U	B^*^27:07:01G	^*^35:03:01G		
PI_B4^¥^	0.333 NM 0.667 P	B^*^40:01:01G	^*^44:02:01G	B^*^40:01:01G	^*^44:02:01G
PI_B5	0.5 NM 0.5 C	B^*^07:02:01G	^*^55:02:01G		
PI_B6	1.0 S	B^*^40:01:01G	^*^56:02:01		
PI_C1	0.5 NM	B^*^44:02:01G	^*^55:02:01G		
PI_C2	U	B^*^52:02:01	^*^52:02:01		
PI_C3	0.25 NM 0.25 C	B^*^44:04	^*^56:02:01		
PI_C4	0.25 NM 0.75 C	B^*^08:156	^*^42:01:01		
PI_C5	0.25 NM 0.75 N	B^*^55:01:01G	^*^55:02:01G		
PI_C6	0.25 NM 0.75 C	B^*^44:02:01G	^*^55:01:01G		
PI_D1	0.5 NM 0.5 C	B^*^15:01:01G	^*^49:01:01G		
PI_D2	0.125 NM 0.875 C	B^*^08:01:01G	^*^44:03:01G		
PI_D3	0.25 NM 0.75 C	B^*^40:01:01G	^*^51:01:01G		
PI_D4^§^	0.5 NM 0.5 C	B^*^13:02:01G	^*^39:01:01G		
PI_D5^§^	0.25 NM 0.5 N 0.25 C	B^*^40:01:01G	^*^40:01:01G		
PI_D6^§^	0.375 NM 0.125 C	B^*^35:60	^*^56:09		
PI_E1	0.5 NM 0.5 C	B^*^35:03:01G	^*^40:01:01G		
PI_E2	0.5 S 0.5 N	B^*^56:02:01	^*^56:02:01		
PI_E4	0.5 NM 0.5 C	B^*^14:02:01G	^*^56:01:01G		
PI_E5	0.5 NM 0.5 S	B^*^39:01:01G	^*^55:01:01G		
PI_F1	0.5 NM 0.5 C	B^*^44:02:01G	^*^50:01:01G		
PI_F2	0.75 NM 0.25 C	B^*^07:02:01G	^*^40:01:01G		
PI_F3	0.125 CM 0.875 C	B^*^18:01:01G	^*^44:02:01G		
PI_F4	0.5 NM 0.5 C	B^*^44:02:01G	^*^43:03:01G		
PI_F5	0.125 NM 0.875 C	B^*^08:01:01G	^*^40:01:01G		
PI_F6	0.25 NM 0.75 C	B^*^35:01:01G	^*^48:01:01G		
PI_G1	0.5 NM 0.5 C	B^*^15:17:01G	^*^37:01:01G		
PI_G2	1.0 U	B^*^07:02:01G	^*^53:17:02		
PI_G4	0.125 NM 0.875 C	B^*^07:02:01G	^*^07:02:01G		
PI_G5	0.25 NM 0.25 C	B^*^35:01:01G	^*^40:01:01G		
PI_G6	0.333 NM 0.583 N 0.083 C	B^*^40:01:01G	^*^44:03:01G		
PI_H1	0.5 NM 0.5 C	B^*^27:10	^*^55:01:01G		
PI_H2	0.5 NM 0.5 C	B^*^07:02:01G	^*^40:10:01G		
PI_H3	1.0 NM	B^*^40:01:01G	^*^48:01:01G		

# *A, African; Ch, Chinese; C, Caucasian; NM, NZ Maori; CM, Cook Island Maori; S, Samoan; N, Niuean; P, others; U, unknown*.

¥ Samples selected for validation.

§ *Samples required manual allele assignment*.

There were 38 HLA-B alleles identified in the 40 Māori and Pacific Island individuals examined. Among these alleles, HLA-B^*^40:01:01 had the highest frequency (28.95%), followed by HLA-B^*^44:02:01 (21.05%) and HLA-B^*^07:02:01 (18.42%). According to the HLA allele frequency database (http://www.allelefrequencies.net), HLA-B^*^40:01:01 is the most prevalent allele in the Han Chinese population (allele frequency of 0.155). It has been reported that the Polynesian people are ancestrally related to Micronesia, Taiwanese Aborigines and East Asia (Kayser et al., 2008; Edinur et al., 2013). This is consistent with our observation on the frequency of HLA-B^*^40:01:01 in this population. On the other hand, the HLA-B^*^44:02:01 and HLA-B^*^07:02:01 alleles are present in multiple populations. When comparing with other studies on Polynesians, we found there were four previously observed alleles not represented in our data (Edinur et al., 2013). Presumably this is because our sample size is insufficient to reflect the full range of HLA-B alleles in Pacific Island or Māori populations. Nevertheless, the most common allele (HLA-B^*^40:01:01) in our study is similar to the Edinur findings, suggesting that this is a common HLA-B allele in this population. Interestingly, there were no HLA-B^*^15:02:01 nor B^*^58:01 observed in the Polynesian individuals. The lack of HLA-B^*^58:01 in Polynesians has previously been reported in other studies (Abbott et al., 2006; Roberts et al., 2015). These alleles have been implicated in carbamazepine and allopurinol-induced severe cutaneous adverse reactions, respectively. However, HLA-B^*^57:01:01, important for abacavir-associated hypersensitivity reactions, was apparent in two samples.

## Discussion

The primary goal of our study was to develop methods for HLA-B class 1 typing on the MinION nanopore sequencer. This study applied PCR across HLA-B exon 2 and exon 3, followed by barcoding and nanopore sequencing of 49 samples simultaneously. The high quality and good depth of coverage of our sequencing data for all samples, including several that were present at low concentration, enabled accurate assignment of HLA-B alleles. With ongoing improvements to the speed, throughput and workflow of MinION flowcells and the associated chemistry, it is likely that multiplexing could be extended to much greater numbers without compromising the ability for accurate typing.

The workflow we employed was straightforward and solely PCR-based. Though the ONT protocol requires 2.0 nM of input amplicon prior to the barcoding step, sample PI_G2 was successfully amplified and produced sequencing data at 0.19 nM, less than 10-fold the recommended amount. That said, even these poorly represented samples generated sufficient data for HLA-B typing. Of all the reads generated from the sequencing device, 199,297 reads had good quality and passed the filters for alignment. Reads were ignored if they did not map to the region of interest. Among those usable reads, varied numbers of reads between samples were observed, ranging from 80 to 17,329 (Figure 4). Regardless of this read-depth variability between samples, adequate coverage was achieved for all samples and allowed for confident HLA-B phasing. It is also worth noting that individual PI_C3 only had 80 reads that were aligned with reference sequences, but alleles could be assigned confidently. Our protocol takes 3 days to complete, comprising 1–1.5 days for library construction, 1 day for sequencing and base calling, and a half-day for data analysis. The sequencing step takes up to 2 days if aiming for more reads; however, it is possible to analyze data prior to the end of the run if desired. It can be argued that the turnaround time of our method is still longer than that of the gold standard SBT method. Other NGS workflows for MinION library construction may take even longer (3–4 days) with the employment of biotinylated probes (Karamitros and Magiorkinis, 2015). Moreover, given the fact that the capacity of the MinION can be enlarged to analyze other HLA loci at high-resolution and more sample input (up to 96 samples at present), MinION-based HLA typing can overcome this limitation. The ability to confidently call the genotype with such a small number of reads suggests that it would be reasonable to increase throughput by sequencing additional HLA loci, or indexing up to 96 individuals per sequencing run.

Our average read length was 1,028 bp indicating that our reads were long enough to cover exon 2 and exon 3 of HLA-B. We are aware that ambiguous typing of HLA-B might occur due to variants outside the region analyzed. This resulted in the assignment of G Codes in several samples (Table 3). In HLA typing, the letter “G” is used to report ambiguous alleles which have identical exon sequences encoding the peptide binding domains (exon 2 and exon 3 for HLA class I). A whole gene sequencing is required to resolve this ambiguity and obtain a full resolution of HLA allele. However, as not all alleles have been completely sequenced, only exon sequences can be mapped in some cases. For example, there are 4,356 HLA-B alleles but only 384 alleles have complete sequence information (Robinson et al., 2014). By this, we mean it is currently more practical to focus on exons solely than to sequence the entire gene for HLA-B typing with minimal ambiguity. Obviously, we are able to increase the read length for complete sequencing of the HLA-B if necessary, as the MinION is capable of very long sequence reads (Carter and Hussain, 2017). Therefore, once all HLA-B alleles in the IMGT/HLA database have full information, our method can be adapted to sequence a full-length HLA-B with greater specificity and sensitivity.

Our results showed that SeqPilot was able to identify HLA-B alleles accurately using the MinION reads. Although the software could not automatically assign HLA-B alleles of all participants (6 cases), it listed the most likely genotype combinations in rank of number of mismatch sites. This enabled us to manually assign HLA-B alleles based on this order. We found that these errors only occurred at nucleotide position 130–136 of exon 2 and on samples which had a (GA)_3_ repeat on one of the allele sequence. Deletion rates of the MinION using R7.3 and R9.0 chemistry are 4.1 and 3.5%, respectively (Jain et al., 2017). Therefore, it may be that deletion errors produced by the MinION device combined with the complex nature of the HLA-B, make accurate interpretation of genotype particularly difficult around this region. Of the six individuals that were manually phased, alleles from three had been identified either by the SBT method or the 1000 Genomes project or both. These alleles were all consistent with our manual assignments, suggesting this approach can be applicable in such circumstances.

The second aim of our study was to examine HLA-B alleles in individuals of Māori and Pacific Island descent living in New Zealand. Previous studies used allele-specific primer PCR for HLA-B genotyping, which provides typing at first field resolution (Edinur et al., 2013; Roberts et al., 2013). Here, we report a feasible method for high-throughput and high-resolution HLA-B typing using NGS. Though ours is a relatively small sample, this initial finding can be used as a reference for future studies on the prevalence of HLA-B in these ethnic groups.

In recent years, various high-throughput HLA typing studies have been conducted using different NGS technologies (Carapito et al., 2016). Though NGS-based HLA typing can be time-consuming (Chua and Ng, 2016), it offers high-resolution, unambiguous, phase-defined HLA alleles, overcoming some of the limitations of traditional approaches. For instance, the current gold standard method (SBT) may generate ambiguous typing due to genotype phase issues and incomplete sequencing. Modern NGS-based HLA typing methods were mostly developed on Illumina MiSeq/HiSeq or PGM Ion Torrent platforms, which employed short-to-medium sequencing read data as an input for HLA allele assignment (Hosomichi et al., 2015). At present, the Illumina platform has been widely adopted due to its high accuracy and high precision for HLA typing. However, the advent of long read single-molecule sequencing holds the promise of achieving full-length phase-defined HLA genes as well as detecting novel and rare variants. An early, very preliminary study suggested the MinION nanopore sequencer has potential for HLA analysis (Ammar et al., 2015). The device and associated chemistry has been continuously improving (Jain et al., 2017). For example, the total error of 2D reads reduced from 9.1% in R7.3 chemistry to 7.3% in R9.0 chemistry (now R9.4). Another advantage of the MinION device is its portability, which raises the possibility of using the device for HLA analysis in field situations or point-of-care settings.

Before this assay could be applied clinically, at least two things would need to occur. First, the nanopore technology is still in a state of relatively rapid development, and the MinION platform would need to stabilize before clinical implementation would be possible. For example, since the work described in this report was completed there have been various further iterations of chemistry and library preparation procedures for the MinION. In addition, newer versions of the nanopore sequencing equipment with higher throughput (such as the GridION and PromethION) have been recently released to the market. Second, a much large study would be required to assess the sensitivity and specificity of the nanopore sequencing and allele calling procedure described in this paper. This would need to be carried out in a cohort which had undergone HLA typing using the current gold standard HLA typing approach of SBT (Erlich, 2012).

## Conclusion

We have described here the development and evaluation of a PCR-based HLA-B sequencing method using MinION Nanopore Technology on R9.4 flow cell. We demonstrated that our method is relatively straightforward and can generate accurate sequencing data from many barcoded samples in a single run. We also reported that precise HLA-B alleles could be obtained from the MinION reads with minimal phase ambiguity. Our protocol can be easily adapted for other HLA loci, or for full gene sequencing, or to employ greater levels of multiplexing. The method could be particularly valuable for research studies examining the role of HLA alleles in ADRs.

## Data availability

The complete sequencing data can be accessed at the NCBI Sequence Read Archive database with the accession number SRP138979.

## Author contributions

KT carried out the laboratory work, data analysis and drafted the manuscript. SC advised on nanopore sequencing procedures and bioinformatic analyses. SG-S contributed to the analysis and assignment of HLA alleles. TM and LS recruited subjects and provided DNA for this analysis. MK supervised the work and contributed to preparation of the manuscript.

### Conflict of interest statement

Author SG-S was employed by JSI medical systems GmbH. The other authors declare that the research was conducted in the absence of any commercial or financial relationships that could be construed as a potential conflict of interest.
